# New insights link low-virulent disc infections to the etiology of severe disc degeneration and Modic changes

**DOI:** 10.2144/fsoa-2019-0022

**Published:** 2019-03-18

**Authors:** Claus Manniche, Søren O'Neill

**Affiliations:** 1 Department of Occupational & Environmental Medicine, Odense University Hospital & Institute of Clinical Research, University of Southern Denmark, 5230 Odense M, Denmark; 2 Spinecenter of Southern Denmark, Lillebaelt Hospital, Institute of Regional Health Research, University of Southern Denmark, 5230 Odense M, Denmark

**Keywords:** bacteria, disc infection, disc inflammation, end-plate damage, Modic changes

## Abstract

Within the last 5 years, international research collaborations including those of several research groups skilled in microbiology, immunology and pathophysiology, have identified a low-virulent intradiscal infection with the ability to provoke gradual and progressive disc degeneration, end-plate disruption, Modic changes and persistent clinical lower-back pain. Certain strains of the *Propionibacterium acne* bacterium seem able to invade, colonize and develop a protective biofilm inside the disc. The interaction of *P. acne*, disc tissues and mononuclear cells of the bone marrow are shown to trigger a relevant immunological response and an ensuing destructive inflammation of the disc and adjacent vertebrae. This process presents on MRI as Modic changes. Recent proof-of-concept data provide compelling evidence for this bacterial disc infection hypothesis.

Modic changes (MC) were first described 30 years ago [1], but over the course of the following 20 years, the phenomenon did not attract much attention. Wider interest was stirred in 2008 when a Danish research team proposed an infection-hypothesis as one of several possible etiologies for MC [2], a hypothesis which proved somewhat controversial. Research activity into disc degeneration and MC has grown considerably in the last decade and more recently, new experimental animal models and laboratory procedures have provided researchers with far more avenues to investigate the phenomenon than only a few years ago. The latest findings suggest that research into MC may soon provide a better and more detailed understanding of the mechanisms and etiologies of at least some chronic discogenic pain. New insights hold out promise that within the foreseeable future, it may be possible to develop new pharmaceutical treatment methods for this subgroup of chronic lower-back pain (LBP) patients. Research into MC may thus prove to be a ‘biological’ keystone in the ‘biopsychosocial’ model, which incorporates valuable insights from immunology and microbiology in addition to the existing radiological and epidemiological research in the area of disc degeneration.

## Definition, pathoanatomy & symptomatology

The literature describes two common types of MC: Type 1 and Type 2. Type 1 is characterized by high signal intensity on T2-weighted MRI scans and low signal intensity on T1 weighted scans. MC Type 2 presents with relatively high signal intensity on both T1 and T2 weighted MRIs. A combination of Type 1 and 2 is often observed within the same mixed lesion. A Type 3 MC has also been described but is rare and of no apparent clinical significance [1,3,4].

Biopsy studies demonstrate that MC Type 1 contain relatively large amounts of granulation tissue and active inflammatory components. Type 2 consists of granulation tissue mixed with fatty tissue and is characterized by relatively low levels of new bone formation [1,4].

In patients with long-standing LBP, the prevalence of MC has been reported in several studies to be approximately 40% by contrast to a prevalence of 6% in the adult background population [5,6]. Also, MC is always observed in relation to a concomitant disc degeneration, whereas disc degeneration without MC is a common finding [7]. A recently published longitudinal twin-cohort study of 438 individuals reported that endplate defects, lumbar disc degeneration and MCs all are independent risk factors for episodes of severe and disabling LBP. Longitudinal analysis showed that MCs followed disc degeneration [8]. Endplate defect has a significant heritability of 55% [8] and clinically, MC are often associated with LBP at all times of the day and nightly pain and morning pain/stiffness are common complaints [9,10].

The MC have been observed in 48% of patients in the aftermath of an acute disc herniation [11] and the literature describes a correlation over time between the extend of the MC and reported pain intensity [12]. MC Type 1 has been shown to be an important prognostic marker for poor clinical outcomes in chronic LBP and is associated with reduced levels of activity [13]. The clear correlation between MC Type 1 and prognosis makes sense in view of other studies that have demonstrated a far greater level of immunological activity in Type 1 compared with Type 2 [14].

## Pathogenesis

### Mechano-immunological pathway

A common cause for the development of MC may be the mechano-immunological pathway, which typically progresses gradually over a longer time period as part of aging. More extensive cases will progress to involve adjacent vertebral endplates that are damaged by mechanical stresses and microfractures. Edema and inflammation in the nearby vertebrae can be the final result of the progressing degeneration, evident on MRI as MC Type 1 and later MC Type 2. Individual factors affect the progression of the condition and varying degrees of pain will develop as the vertebral endplate is richly innervated by free nerve endings which may be sensitized and stimulated by inflammatory mediators [15].

### Infectious pathway

A disc herniation or tears in the annulus fibrosis provides an infectious route by which bacteria may migrate from the bloodstream to the inside of the disc. Once inside the relatively cocooned internal disc material, the bacteria may proliferate, produce a self-protective biofilm and induce an inflammatory reaction with ensuing tissue damage [16,17]. The mechano-immunological and infectious pathways are probably involved concomitantly in many cases, which is likely to accelerate tissue damage in disc, vertebral endplates and corporae [18]. See below.

## The infectious pathway, two necessary preconditions

### A ‘leaky’ disc

From an early embryological stage, the internal tissues of the intervertebral disc are immunologically sequestered. The avascular nucleus pulposis is laterally encapsulated by the only superficially vasculated annulus fibrosis and superiorly/inferiorly by the cartilagenous and osseos vertebral endplates [18]. At birth, the internal disc tissues are thus avascular and perfusion of nutrients and metabolites are accomplished by passive and mechanically assisted diffusion. As a consequence, the nucleus is immunologically isolated from other body tissues. In other words, the nucleus is from birth without immune response competence [19].

This is underlined by studies which demonstrate that nucleus material from an intact disc mixed with *Propionibacterium acne* bacteria fails to produce an immunological response, whereas nucleus material from a ruptured disc combined with *P. acne* induces an appropriate immune response [19].

In cases of an acute disc herniation, a sudden rupture of the annulus fibrosis or the hyaline-cartilagenous endplate leads to prolapse of the disc tissue which may involve a small avulsion fracture of a fragment of the endplate and/or the annulus [20,21]. As end-plate cartilage only regenerates poorly, if at all, a persistent tissue loss is to be expected – often a 3–8 mm lesion in the hyaline endplate remains [20]. As a consequence, the endplate fails to function as an effective barrier between the disc and vertebrae [22], thus increasing the risk of intravertebral edema, in other words Modic changes.

Furthermore, as a complication to the structural disruption, immunologically active components such as polymorphonuclear leucocytes can enter the disc material from other tissues. Over time, this will result in the ability to generate a relevant immune response within the disc tissues when heterotopic substance or microorganisms invade the disk [23,24]. Ultimately, the nucleus becomes immunologically competent and is capable, for better or worse, of mounting an immunological response to bacteria, commonly *P. acne*. This intradiscal immune response involves TNF-α, IL1, IL6 and IL8 [23,24]. The immune response also leads to concomitant destruction of intradiscal tissue and the adjacent intervertebral hyaline endplate. The result, in some cases, is a severe and progressive degeneration of the disc and endplate and pronounced inflammation in the vertebral bone marrow [23,25,26]. MC Type 1 represents the radiological evidence of this process on MRI and clinically the condition is typically associated with pain and stiffness [9,10].

### Bacteria within the disc

Biopsy studies of bacterial infection in human disc material confirm that *P. acne* is the most commonly found microorganism in disc tissue [27,28]. In rat models, rabbits and human tissues, experimental studies show that the bacterium can initiate a degeneration of the disc with ensuing damage to the endplates and development of MC [25–27]. *P. acne* is a 1–2 μm long, rod-shaped bacterium, generally considered relatively harmless and ubiquitous on the skin and in the oral cavity. It is an anaerobic organism but can survive briefly in aerobe environments. It is known to cause acne vulgaris on the skin and dental abscesses in the mouth [28].

In broader terms, *P. acne* is a group of almost 100 bacterial subclasses with a preference for skin and mouth and the virulence between subclasses can vary considerably. The ability to spread, colonize and develop a protective biofilm is considered important determinants of such virulence. The biofilm is a thin, slimy layer of filament-like structures in an amorphous substance, which serves to bind the bacteria together, to function as a medium for chemical communication between them and to provide protection against the external environment such as assaults from the immune system. Furthermore, *P. acne* can produce hyaluronidase, propionic acid and lipases which increase their virulence. *P. acne* has been associated with a number of diseases of the prostate (cancer), lungs (sarcoidosis) and brain (Parkinson's disease) as well as being a potential source of chronic infection in arthrodesis [29].

The avascular conditions within the intervertebral disc, combined with the protective barrier of the biofilm formed by aggregations of 100–200 bacteria, provides an optimal milieu of low PH and low oxygen levels. Conversely, the environment of the adjacent vertebral bone marrow is not conducive for bacterial growth [30].

Furthermore, the *P. acne* bacterium can downregulate its metabolism and survive in a state hibernation or low activity for prolonged periods of time, presumably years, which increases its resistance to antibacterial drugs.

**Figure 1.**
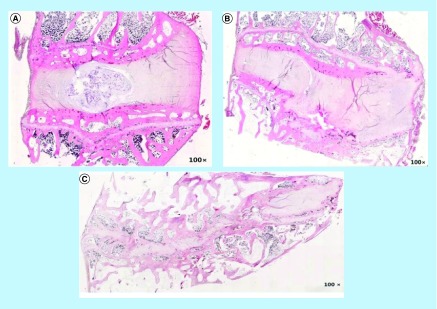
**Hematoxylin and eosin staining of rabbit discs 8 weeks following inoculation with *Propionibacterium acne/Staphylococcus aureus*.** **(A)** Isotonic sterile saline. Intact nucleus/annulus and endplates. **(B)** *P. acne*. Nuclear degeneration, annular disruption and end-plate fracture. **(C)** *S. aureus*. extensive disc disruption, narrowed intervertebral space, ill-define endplate borders. Reproduced with permission from [25].

Recent data from experimental animal studies demonstrate that inoculation with active *P. acne* into the intervertebral discs of rabbits and rats, results in a gradual development of disc degeneration, end-plate damage and intravertebral edema over a period of a few weeks (Figure 1) [23,25]. The MRI scans demonstrated the development of MC Type 1 in one of these studies. By contrast, injection of *Staphylococcus aureus* bacterium was shown to rapidly develop into a pronounced spondylodiscitis [25]. The investigators concluded that *P. acne* is capable of instigating disc- and end-plate degeneration in animal models and that the resulting MRI findings mirror MC as seen in patients.

## The infectious pathway in the disc: step by step

The most recent research as cited here can be summarized as the following steps toward the pathogenesis of infectious degeneration, end-plate destruction and development of MC:

- The intervertebral disc undergoes normal, age-related degeneration with concomitant mechanical susceptibility for sprain injuries;
- Immunological cross talk with other tissues, once exposed to the circulating immune system;
- Development of intradiscal immunological competence;
- Invasion of bacteria, most commonly *P. acne*;
- Proliferation, colonization and biofilm development of bacterial aggregates within the disc;
- Low-grade immune response reaction within disc;
- Gradually progressing, immunologically mediated inflammation and disruption of the discal tissue and adjacent vertebral endplate;
- Compromise of the barrier-effect of the vertebral endplate;
- Immunological cross talk between the infected disc and adjacent vertebral bone marrow;
- Development of radiologically evident MC and clinical pain.

## The infectious pathway: proof of concept

As described above, the cascade of events from age-related degeneration and mechanical compromise of the disc to infection and MCs, constitutes a number of separate but interrelated processes. In addition to these, a number of individual factors are likely to influence which patients are affected, what tissues are involved, how pronounced and how rapid the condition progresses and so on. This in turn will influence the clinical consequences of the condition in terms of tissue damage and pain.

The infectious etiology and pathway of MCs have been hotly debated in recent years [31,32]. It is generally acknowledged that obtaining sterile biopsies from disc and vertebrae is technically challenging, as *P. acne* will be present in hair follicles and sweat glands despite strenuous efforts to sterilize the skin. The risk of cross contamination between the skin and biopsy material can be minimized, but is always present, which may lead to incorrect test results and overestimation of the prevalence of *P. acne* in degenerated discs [33]. Also, the methodological challenges associated with analysis of disc biopsy material once extracted are not insignificant [34]; for example, incorrect (an)aerobic handling of the bacterial cultures may result in underestimation of the prevalence of infection. Generally, there is no consensus on the most appropriate methodology.

All in all, the points raised above have led to a situation where contradictory data on the prevalence of *P. acne* in disc biopsies have been published, ranging from 0 to 55% of sampled discs, and consequently researchers in the field have drawn different conclusions based on the literature. Thus far, the variation between studies has been best explained by these methodological challenges. Recent proof-of-concept data from the USA and Denmark, however, provide compelling evidence for the bacterial disc infection hypothesis, which in turn, should move the focus of attention from methodological issues to a more fundamental discussion of the phenomenon of MCs [34,35].

**Figure 2.**
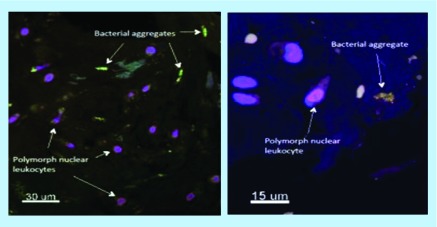
**Microscopic tissue sample stained in the flourescence *in situ* hybridization method.** Biopsy tissue from herniated disc tissue retrieved during surgery. Biofilm (green) covered aggregates of *Propionibacterium acnes* bacteria. The close presence of pylomorphonuclear leucocytes (lilac) indicate inflammatory immune response. Reproduced with permission from John Wiley/Rightslink (2018) [35].

Using the fluorescence *in situ* hybridization (FISH) method in patients with confirmed disc herniation, where a biopsy is extracted during surgery, the presence of *P. acne* has been demonstrated in tissues of patients, who otherwise showed no signs of infectious disease (Figure 2) [35].

The benefit of the FISH methodology is that it can demonstrate the presence of *P. acne in situ* within the disc material. Specifically, as biofilm covered aggregates of bacteria with clusters of polymorphonuclear leukocytes, which is an indicator that the bacterial aggregates have settled within the tissue and been present for long enough to provoke an inflammatory reaction mediated by leucocytes [35]. The intradiscal morphology illustrated in Figure 2 indicates that this biofilm growth provokes a pathological reaction and satisfies the classical definition of an active infection. It contradicts the presence of *P. acne* in biopsy material as only cross contamination. In this manner, the FISH methodology provides the basis for more solid conclusions than previous investigations employing PCR techniques and microbial culturing methods. In these other techniques, the original tissue structure is destroyed in the analytical process, whereas the original morphology of the disc tissue is preserved in FISH analysis. The prevalence of MCs is still unclear, however, as only two FISH studies has been published.

## Treatment

In most cases, the treatment of back pain associated with MCs is the same as recommended by clinical guidelines for back pain with disc degeneration/spondylosis in general. However, in many cases of MC, and in contrast to back pain in general, no improvement is seen even after a prolonged period of exercise therapy and physical activity [36]. There is no general consensus on the most effective pharmaceutical therapy, but a few studies have demonstrated a short-term effect of steroid injections, biphosphonates and anti-TNF-α antibodies [37]. If a low-virulent infection of the disc is suspected (Box 1), a course of antibiotics is a reasonable clinical option if other treatment forms have been tried without satisfactory results. In 2013, a well-designed RCT with a 1-year follow-up period demonstrated clinical effects of a 3-month course of amoxicillin/clavulanic acid in patients with disc herniation and MC [38]. More than 60% of patients in the active treatment group reported clinically and statistically significant improvements in pain and function over the length of the follow-up period. A later randomised clinical trial (RCT) has confirmed clinical improvements with antibiotic treatment of MC [39] and further RCTs are underway for publication by 2019.

## Perspectives for future management

With the presented evidence for an infectious pathway as an additional etiology of MCs in mind, it is salient to consider the following points:

- Can reliable *in vivo* methods of differential diagnostics of the infectious pathway in contrast to the mechano-immunological pathway be developed?
- How prevalent is the infectious pathway as a clinically important phenomenon?
- Is the infectious pathway to MCs preventable?
- Can patients be identified for whom anti-inflammatory treatment as opposed to anti-microbial treatment, is relevant?
- Which management protocols are most effective and associated with least complications for the patient and the environment?

For future research, it is important to arrive at an international consensus on which analytical methods are most appropriate for assessing the presence of microorganisms in MC (e.g., PCR or *in vitro* culturing) and these methods must take into consideration the tendency for *P. acne* to aggregate into biofilm-covered clusters unevenly distributed throughout infected tissues. Capoor *et al.* [34] suggests that future PCR methods should assess whether the number of microorganisms is sufficiently large to be considered indicative of an actual infection. They suggest that a lower limit of ‘tissue burden’ be established (e.g., 1000 CFU/g tissue) under which the presence of bacterial DNA cannot be considered indicative of active infection [16]. The use of the FISH method should also be standardized and employed routinely when the bacteria in question have a predilection for clustering in biofilm covered aggregates.

In relation to imaging, work is being done to improve MRI and PET-CT methods to better distinguish between the different types of vertebral edema. Currently, the manner in which MC Type 1 and 2 are described varies considerably and there is no general consensus on how to standardize reports of MC [40]. Also, *in vivo* NMR spectroscopy is actively being developed in an attempt to demonstrate the presence of propionic acid, one of the known metabolites of the *P. acne* bacterium. Such methods may in future distinguish between MC of mechano-immunological etiology and those on an infectious basis [41].

In terms of prevention, prophylactic treatment with antibiotics following an acute disc herniation, disc surgery or other causes of disc rupture/perforation may prove effective in preventing an opportunistic *P. acne* infection and more RCTs of antibiotics and anti-inflammatory medication are warranted to answer: which management approaches provides the best clinical outcomes with the fewest complications and side effects?

## Conclusion

With a new, wider scientific foundation of intradiscal bacteria invasion, development of disc degeneration, end-plate damage and Modic changes, we may be hopeful that in the coming decades, the individual pieces will come together to provide a more detailed picture of this complex phenomena. The new insights represent an exciting basis for further research and potentially the development of new management approaches based on antibacterial and anti-inflammatory pharmaceuticals for a subgroup of patients with persistent LBP.

**Box 1.**  **How to diagnose & treat persistent back pain where low-virulent bacterial infection is suspected**

Patient with persistent and well-localized back pain. Exacerbation with exercise therapy.

Awakens during the night with pain and reports stiffness for more than 30 min in the morning.

History suggests a disc herniation within the previous 2–3 years, after which sciatica improved, but back pain persisted or increased.

Various relevant conservative treatments were explored without satisfactory effect. Average pain intensity > 4/10.

MRI: Modic change Type 1 or 1/2 in the relevant painful region. Consider antibiotics (general practitioner [GP] or rheumatologist) if other treatment forms have been applied with successful results.

If treatment is offered: Bioclavid 500 mg × 3/day for 100 days.

Pay attention to common side effects/complications: allergic reaction, diarrhea, fatigue, fungal infections, elevated liver-enzymes, etc.

This is not a standardized treatment protocol and treatment should be followed closely by the responsible doctor. Treatment should only be offered after careful consideration of the individual Modic change case, where pain is persistent, function is significantly affected and other relevant conservative options have been explored without sufficient effect.

Note: Effects of antibiotics for Modic changes will likely only manifest after 2–4 months. Slow improvements for following 12 months are often.

**Executive summary**

- In patients with long-standing lower back pain (LBP), the prevalence of Modic changes (MC) has been reported in several studies to be higher than 40%.
- The MC has been observed in 48% of patients following an acute disc herniation.
- The MC Type 1 has been shown to be an important prognostic marker for poor clinical outcomes in chronic LBP.
- The disc nucleus is without immune response competence from birth.
- When microorganisms invade leaky discs, the discs may overtime develop the ability to initiate a relevant immune response.
- *Propionibacterium acnes* – a relatively low-virulent, rod-shaped bacterium with a preference for skin and the oral cavity has the ability to spread, colonize and develop a protective biofilm inside the disc.
- Immune responses to invading bacteria may lead to concomitant destruction of intradiscal tissue and the adjacent intervertebral hyaline endplate, visualized on magnetic resonance (MR) as MC.
- Using the fluorescence *in situ* hybridization (FISH) method in patients with disc herniation, biopsies have demonstrated the presence of *P. acne* biofilm and inflammation in disc tissues of patients, who otherwise showed no signs of infections.
- These new insights represent an exciting basis for further research and potentially the development of new management strategies based on antibacterial and anti-inflammatory pharmaceuticals for a subgroup of patients with LBP.
- If a low-virulent infection of the disc is suspected, a course of antibiotics is a reasonable option if other treatment forms have been applied without satisfactory results.
